# Resistance of SARS-CoV-2 Omicron variant to convalescent and CoronaVac vaccine plasma

**DOI:** 10.1080/22221751.2022.2027219

**Published:** 2022-01-28

**Authors:** Yingdan Wang, Yunping Ma, Yan Xu, Jiangyan Liu, Xiang Li, Yuyuan Chen, Yan Chen, Jun Xie, Lianbo Xiao, Zheng Xiang, Fan Wu, Jinghe Huang

**Affiliations:** a Key Laboratory of Medical Molecular Virology (MOE/NHC/CAMS) and Shanghai Institute of Infectious Disease and Biosecurity, Shanghai Public Health Clinical Center, School of Basic Medical Sciences, Fudan University, Shanghai, People’s Republic of China; b The Biomedical Translational Research Institute, Faculty of Medical Science, Jinan University, Guangzhou, People’s Republic of China; c Guangdong Provincial Key Laboratory of Tumor Interventional Diagnosis and Treatment, Zhuhai Institute of Translational Medicine, Zhuhai People’s Hospital Affiliated with Jinan University, Jinan University, Zhuhai, People’s Republic of China; d Department of Joint Orthopedics, Guanghua Hospital Affiliated to Shanghai, University of Traditional Chinese Medicine, Shanghai, People’s Republic of China; e Department of Paediatrics and Adolescent Medicine, Li Ka Shing Faculty of Medicine, The University of Hong Kong, Hong Kong SAR, People’s Republic of China

To the Editor: Vaccination is considered as an effective method to prevent SARS-CoV-2 infections and control the pandemic of COVID-19. Clinical studies revealed that the SARS-CoV-2 vaccines have reassuring safety and successfully reduced COVID-19 cases and related morbidity and mortality [1,2]. However, the appearance of SARS-CoV-2 variants that were resistant to immune responses may reduce the efficacy of current SARS-CoV-2 vaccines. Recently, WHO classified a newly emerging SARS-CoV-2 variant Omicron (B.1.1.529) as a variant of concern (VOC) [3]. Compared with the original SARS-CoV-2 and other VOC, this variant has more than 30 mutations on its spike, including some substitutions such as E484A, N501Y, D614G which are able to increase viral transmission and resistant to neutralization [4]. Furthermore, the infections of Omicron variant were also detected from patients who recovered from previous SARS-CoV-2 infection, suggesting reduced protection from prior infections.

Here, we generated pseudoviruses carrying the spike protein of Omicron, Alpha, Beta, Gamma, Delta, Lambda, and Mu mutants (Table 1). We evaluated the sensitivity of these variants to the neutralizing antibodies induced by prior infections and two doses of inactivated vaccine (CoronaVac).

**Table 1.** Mutation sites of Alpha, Beta, Gamma, Delta, Lambda, Mu, and Omicron.

VOC/VOI	Mutation sites
Alpha	Δ69-70, Δ144, N501Y, A570D, D614G, P681H, T716I, S982A, and D1118H
Beta	D80A, D215G, Δ241-243, K417N, E484K, N501Y, D614G, and A701V
Gamma	L18F, T20N, P26S, D138Y, R190S, K417T, E484K, N501Y, D614G, H655Y, T1027I, and V1176F
Delta	T19R, Δ157-158, L452R, T478K, D614G, P681R, and D950N
Lambda	G75V, T76I, Δ247-253, L452Q, F490S, D614G, and T859N
Mu	T95I, Y144S, Y145N, R346K, E484K, N501Y, D614G, P681H, and D950N
Omicron	A67V, Δ69-70, T95I, G142D, Δ143-145, Δ211, L212I, ins214EPE, G339D, S371L, S373P, S375F, K417N, N440K, G446S, S477N, T478K, E484A, Q493R, G496S, Q498R, N501Y, Y505H, T547K, D614G, H655Y, N679K, P681H, N764K, D796Y, N856K, Q954H, N969K, and L981F

Sixteen convalescent plasma samples were collected from recovered patients of COVID-19 on the day of discharge from January to March 2020 in Shanghai, China [5]. Neutralization assay revealed that 16 convalescent plasma samples showed an average 10.5-fold reduction of neutralization against Omicron variant when compared with the SARS-CoV-2 WT (Figure 1(A)), and 2.2, 5.4, 4.8, 2.6, 1.9, and 7.5-fold reduction in neutralizing Alpha, Beta, Gamma, Delta, Lambda, and Mu variants, respectively.

**Figure 1.**
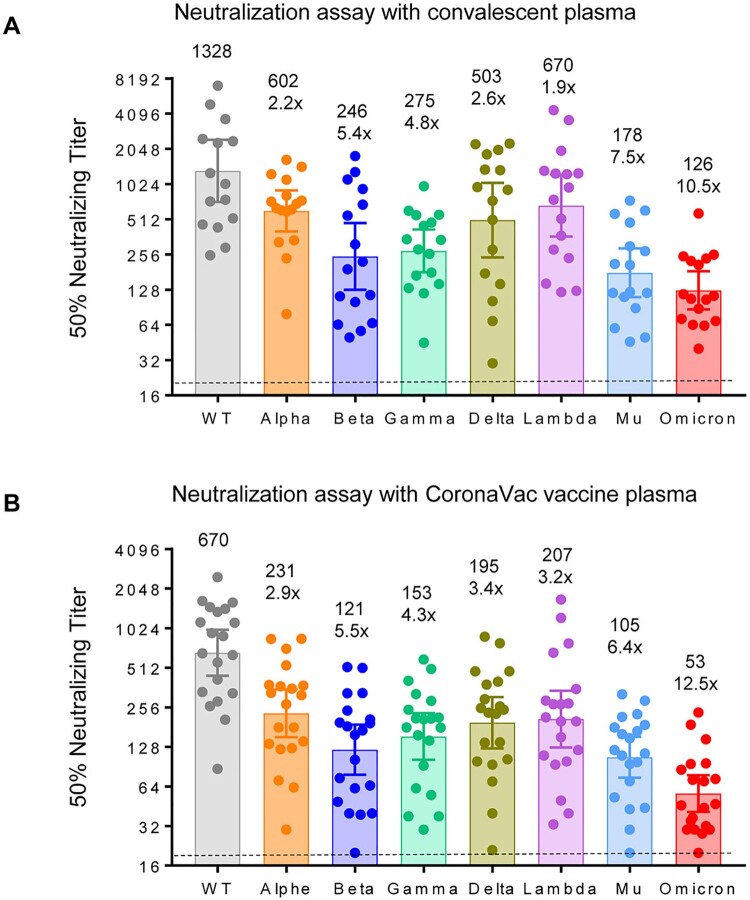
Neutralization efficacy of inactivated vaccine plasma and convalescent plasma.

To compare the sensitivity of the parental and the variants to the neutralizing antibodies induced by vaccination, we chose 20 representative plasma from ConoVac recipients with diversified neutralizing antibody titres from low to high but excluded the plasma with titres below 40 against wild-type strain. All the plasma samples were collected on day 14 after the second dose of ConoVac from May to June 2021. Twenty plasma from CoronaVac vaccine recipients showed an average 12.5-fold reduction in neutralizing Omicron variant (Figure 1(B)), 2.9, 5.5, 4.3, 3.4, 3.2, and 6.4-fold reduction in neutralizing Alpha, Beta, Gamma, Delta, Lambda, and Mu variants, respectively.

## Discussion

There have been over 8.2 billion doses of SARS-CoV-2 vaccines administrated in more than 4.3 billion persons around the world [1]. However, the emerging of the Omicron variant raises serious concern since it escapes the majority of SARS-CoV-2 neutralizing antibodies [6], significantly decreases the immune protection elicited by the existing COVID-19 infection and mRNA vaccines [7–9]. Similarly, Zhang et al. tested 28 serum samples from COVID-19 convalescent patients and observed an 8.4-fold drop in neutralization against Omicron variant compared with the D614G reference strain [10]. Consistent with these reports, we observed 10.5-fold decrease but still detectable neutralization (average GMT 126) against Omicron variant in convalescent plasma from recovered patients of COVID-19. To be mentioned, the convalescent plasma that we evaluated in this report were collected at the time of discharge, which are at the peak level of neutralizing antibodies. Since it has been reported that the SARS-CoV-2-specific neutralizing antibodies would wane after recovery [11], whether the individuals who had prior infection are susceptible to Omicron variant should be carefully evaluated.

We also observed about 12.5-fold decrease of neutralization against Omicron variant from recipients who received two doses of inactivated vaccine. It is better than the previous reports about two doses of mRNA vaccines in which 22- and 180-fold decrease of neutralization was observed in Pfizer-BNT vaccinated recipients [7,8]. However, the difference may be due to different assays or sample time. Previous reports have indicated that booster vaccinations with 3rd dose of vaccines greatly increase vaccine efficacy [12,13]. Whether the booster vaccinations with 3rd dose of mRNA or inactivated vaccine provide sufficient protection against Omicron variant should be evaluated.

Comparing with previous naturally occurring SARS-CoV-2 variants including Alpha, Beta, Gamma, Delta, Lambda, and Mu, Omicron variant exhibits an unprecedented degree of immune escape of the neutralization from prior infections and vaccination by two doses of CoronaVac. Although the Mu variant (a variant of interest) was the most resistant variant to date [14], the Omicron variant was 1.4- and 2.0-fold as resistant to neutralization by convalescent plasma and vaccine plasma, respectively, as the Mu variant. Omicron variant was 2.0- and 2.3-fold as resistant to neutralization by convalescent plasma and vaccine plasma, respectively, as the beta variant.

Since the Omicron variant has been detected in over 100 countries, a comprehensive head-to-head evaluation of current vaccine strategies against Omicron variant, and update of vaccine components and therapeutics antibodies may be required to catch up with the circulation of this variant.

## Materials and methods

### Plasma samples

Plasma samples from convalescent COVID-19 patients were collected from Shanghai Public Health Clinical Center from January to March 2020 on the day of discharge. This study was conducted under a clinical protocol approved by the Investigational Review Board (IRB) of the Shanghai Public Health Clinical Center (Study number YJ-2020-S021-01). Plasma samples from CoronaVac recipients who received two doses of inactivated vaccine were collected from GuangHua Hospital Affiliated to Shanghai from May to June 2021 two weeks after the second vaccination. Ethical approval was obtained from the Shanghai GuangHua Hospital (2020-K-115). All participants signed an informed consent approved by the IRB.

### Pseudovirus neutralization assay

Genes of the Omicron (GISAID: EPI_ISL_6590782.2), SARS-CoV-2 (NC_045512), Alpha, Beta, Gamma, Delta, Lambda, and Mu of SARS-CoV-2 spike protein were codon-optimized and synthesized by Genscript and constructed in the pcDNA3.1 vector. Pseudoviruses were generated by co-transfection of 293T cells with the spike protein expression plasmids and the pNL4-3.Luc.R-E- backbone.

Neutralization activity of plasma from COVID-19 patients and vaccine recipients was measured using a single-round pseudovirus infection of Huh-7 cells [1]. 1 × 10^4^ Huh-7 cells were seeded in a 96-well plate and cultured for 12 h. Then, plasma was four-fold serially diluted from 1:20 and mixed with pseudovirus for 1 h. The mixture was added to cultured Huh-7 for infection. The culture medium was refreshed after 12 h and incubated for an additional 48 h. Assays were developed with a luciferase assay system (Promega), and the relative light units (RLU) were read on a luminometer (Perkin Elmer). The plasma titres were calculated as NT50 and expressed as the highest dilution of plasma which results in a 50% reduction of luciferase luminescence compared with virus control. Statistical analyses were carried out using GraphPad Prism 7.0.
